# A Target Animal Effectiveness Study on Adjuvant Peptide-Based Vaccination in Dogs with Non-Metastatic Appendicular Osteosarcoma Undergoing Amputation and Chemotherapy

**DOI:** 10.3390/cancers14051347

**Published:** 2022-03-06

**Authors:** Laura Marconato, Alessia Melacarne, Marina Aralla, Silvia Sabattini, Luca Tiraboschi, Valentina Ferrari, Offer Zeira, Andrea Balboni, Eugenio Faroni, Dina Guerra, Luciano Pisoni, Erica Ghezzi, Letizia Pettinari, Maria Rescigno

**Affiliations:** 1 Department of Veterinary Medical Sciences, Alma Mater Studiorum University of Bologna, 40064 Bologna, Italy; silvia.sabattini@unibo.it (S.S.); a.balboni@unibo.it (A.B.); eugenio.faroni@unibo.it (E.F.); dina.guerra2@unibo.it (D.G.); luciano.pisoni@unibo.it (L.P.); 2 IRCCS Humanitas Research Hospital, 20089 Milan, Italy; alessia.melacarne@hunimed.eu (A.M.); luca.tiraboschi@humanitasresearch.it (L.T.); maria.rescigno@hunimed.eu (M.R.); 3 Pronto Soccorso Veterinario Laudense, 26900 Lodi, Italy; marina.aralla@gmail.com; 4 Department of Biomedical Sciences, Humanitas University, 20090 Milan, Italy; valentina.ferrari@humanitasresearch.it; 5 San Michele Veterinary Hospital, 26900 Lodi, Italy; offer@ospedalesanmichele.it (O.Z.); erica.ghezzi@gmail.com (E.G.); letizia.pettinari@gmail.com (L.P.)

**Keywords:** translational research, osteosarcoma, dog, cancer vaccine

## Abstract

**Simple Summary:**

Despite efforts to develop novel treatment strategies, human and canine osteosarcomas continue to have limited overall survival. Spontaneous canine osteosarcoma shares many molecular similarities with humans, and shows the same aggressive disease course, thereby rendering the dog an effective model for the human disease equivalent. In both species, surgery followed by chemotherapy represents the gold standard treatment. Immunotherapy represents a promising treatment modality. A peptide-based anticancer vaccine was administered to 20 dogs with non-metastatic osteosarcoma as an add-on therapy to standard treatment consisting of limb amputation and adjuvant chemotherapy. Endpoints were to evaluate the efficacy and safety of this combined therapeutic approach. By using a bacterial-based strategy for vaccine development, we report an efficacious induction of an immune response, ultimately translating in improved outcome compared with historical controls receiving standard-of-care treatment. The results of this clinical trial provide promising potential for future management in both humans and dogs with osteosarcoma.

**Abstract:**

Despite efforts to develop novel treatment strategies, human and canine osteosarcomas continue to have poor prognosis and limited overall survival. The aim of this clinical trial was to test the antitumor effect and safety of multiple dermal administrations of a peptide-based anticancer vaccine in dogs with non-metastatic appendicular osteosarcoma undergoing standard of care (SOC), consisting of limb amputation and adjuvant chemotherapy. Salmonella-infected canine osteosarcoma cells were induced to release immunogenic peptides in the extracellular space via Cx43 hemichannels opening; the secretome was collected and constituted the vaccine. Dogs with non-metastatic appendicular osteosarcoma were eligible for recruitment. Following limb amputation and adjuvant carboplatin, dogs were vaccinated on a monthly basis for six times and followed up with serial thoracic radiographs. A population of dogs undergoing SOC treatment (amputation and adjuvant carboplatin) before the vaccine was available served as controls. Primary endpoints were time to metastasis (TTM) and tumor-specific survival (TSS). Secondary endpoints were feasibility, toxicity, T-cell and humoral immune responses. A total of 20 dogs were vaccinated along with SOC and 34 received SOC only. Vaccine-specific humoral and T-cell responses were observed; their amplitude correlated with TSS. Vaccine-associated toxicity was not recorded. TTM and TSS were significantly longer in vaccinated versus unvaccinated dogs (TTM: 308 vs. 240 days, respectively; *p* = 0.010; TSS: 621 vs. 278 days, respectively; *p* = 0.002). In dogs with non-metastatic osteosarcoma undergoing SOC, the addition of a bacteria-based vaccination strategy increased TTM, thereby prolonging survival, while maintaining a safe profile. Additionally, vaccinated dogs developed a long-term tumor-specific response, as documented by the immunomonitoring of these patients over time. These results hold promise for future management of canine osteosarcoma.

## 1. Introduction

Appendicular osteosarcoma is the most frequent and aggressive bone tumor in dogs, with a low long-term survival rate. Even with the current multimodal treatment, which is based on limb amputation and postoperative dose-intense chemotherapy, the 2-year survival rate is approximately 10–26%, with the greatest majority of dogs dying because of distant metastatic disease [1,2]. This poor survival underscores the urgent need for alternative effective therapeutic approaches.

Immunotherapy, currently considered to all intents and purposes the fourth strategy in the fight against cancer, alongside surgery, radiotherapy, and chemotherapy, aims at instructing or reactivating the immune system to recognize tumor cells as foreign, ultimately eliminating them. In recent years, immunotherapy has been shown to be effective for treating pediatric sarcomas [3], and its indication is continually increasing.

Osteosarcoma is considered to be immunogenic, thereby being potentially targetable by the immune system. The first report supporting this theory dates back to the documentation of spontaneous regression of osteosarcoma in four dogs [4]. Subsequently, it was observed that dogs contracting bacterial infections after limb-sparing surgery had a prolonged survival, hypothesizing an antitumor role by the immune system [5,6,7].

The effect of the immune system was also evaluated in mouse models of osteosarcoma with chronic bacterial osteomyelitis [8]. The activation of the innate immune system and, in particular, of natural-killer cells and macrophages counteracted tumor neoangiogenesis, significantly prolonging survival time.

Liposomal muramyl tripeptide phosphatidyl ethanolamine (L-MTP-PE) is an immunotherapeutic agent that activates macrophages and monocytes, triggering the release of pro-inflammatory cytokines that contribute to the tumoricidal effect [9]. In dogs with osteosarcoma, L-MTP-PE showed antimetastatic activity [10].

More recently, the anticancer efficacy of an attenuated therapeutic vaccine called ADXS31–164, consisting of recombinant Listeria monocytogenes expressing a human chimeric protein HER2/neu, has been evaluated in dogs with osteosarcoma, resulting in a longer disease-free interval and survival compared with standard of care (SOC) treatment [11].

It has been shown that Salmonella infections of human, murine, and canine tumor cells promote the release of non-conventional ER-stress-response-derived immunogenic peptides (ERstrePs) by connexin43 (Cx43)-hemichannels, thereby inducing an antitumor response in these model systems [12]. A previous clinical study by our group documented that a heterologous, peptide-based anticancer vaccine induced the expansion of tumor specific CD8+ T-cells in dogs with sarcoma, without any reported toxicity [12].

Based on these considerations, the focus of the present clinical trial was to test the antitumor effect and safety of multiple dermal administrations of a peptide-based anticancer vaccine in dogs with non-metastatic appendicular osteosarcoma undergoing SOC. Additionally, because canine osteosarcoma has a similar clinical presentation, tumor biology, and histopathological appearance to its human counterpart, as well as dysregulation of key molecular pathways [13,14], the dog has been proposed as a possible model for the human tumor, allowing for shorter data maturation time and holding translational relevance for new therapeutic approaches.

## 2. Materials and Methods

### 2.1. Trial Design

This was a multi-center, single-arm, open-label prospective trial of a peptide-based anticancer vaccine in dogs with newly diagnosed, non-metastatic osteosarcoma presented to the University of Bologna, Ospedale San Michele, and Pronto Soccorso Veterinario Laudense. The Ethical Committee of the University Veterinary Hospital (Bologna, Italy) approved the study (protocol 7641); written consent for entry into the trial was obtained from all dog owners. The vaccine was provided free of charge, whereas dog owners were responsible for all other medical expenses.

Client-owned dogs of any age, sex, or breed with histologically proven osteosarcoma of the appendicular skeleton were considered for inclusion. Dogs were required to undergo a complete staging work-up, consisting of history and physical examination, complete blood cell count with differential, serum biochemistry profile, urinalysis, thoracic radiographs, and abdominal ultrasound, or total body CT scan (TBCT), and only dogs free from metastatic disease were eligible for recruitment. After limb amputation and regional lymphadenectomy, dogs had to be treated with carboplatin at the dose of 300 mg/m^2^ IV every 3 weeks for 4 cycles. Following the third carboplatin administration, dogs were ultimately allowed to initiate immunotherapy if they were confirmed free of macroscopic metastatic disease through the conductance of physical examination and TBCT, or thoracic radiography plus abdominal ultrasound.

Dogs were not enrolled if they had previously undergone chemotherapy or radiation therapy, if they had evidence of metastasis at presentation or developed metastasis during the first three chemotherapy cycles, or if they had clinically relevant comorbidities that would limit their expected lifespan. Treatment with nonsteroidal anti-inflammatory drugs or other analgesics was allowed before enrollment and during the trial. Finally, dogs were also excluded if osteosarcoma involved the axial skeleton, metacarpal/metatarsal, or digital bones, due to the different biological behavior [15].

### 2.2. Vaccine Preparation

Primary canine osteosarcoma cells (either autologous or heterologous) were obtained from dissociation of dog tumor samples. Tissues were minced with a scalpel in a cell strainer and the derived single cell suspension was washed and filtered with a cell strainer (100 µm). Cells were counted and cultured at high concentration in Dulbecco’s Modified Eagle Medium (DMEM) supplemented with 10% fetal bovine serum (FBS) United States (U.S.) origin, 2 mM glutamine, and 1x penicillin–streptomycin solution (complete medium). All heterologous vaccines were generated starting from the same primary osteosarcoma cell line (“universal donor cell line”).

Vivofit^®^ (Typhoid vaccine live oral Ty21a) is a vaccine containing the attenuated strain of Salmonella enterica serovar typhi Ty21a and is grown at 37 °C in Luria broth.

The vaccine was prepared as previously described (12). Single bacterial colonies were grown overnight and restarted the next day to reach an absorbance at 600 nm of 0.6, corresponding to 0.6 × 10^9^ colony-forming units (CFUs)/mL. Canine osteosarcoma cells (2 × 10^6^ cells/mL) were incubated with the bacteria for 90 min, in tubes, at a cell-to-bacteria ratio of 1:50, in the appropriate medium containing L-glutamine without antibiotics. The cells were washed and incubated in medium supplemented with gentamicin (50 mg/mL), in tubes, for 18 h to kill extracellular bacteria.

During incubation, immunogenic peptides are released via Cx43 hemichannels by cancer cells in the extracellular space; the supernatant enriched with immunogenic peptides (secretome) was collected and filtered through a 0.22 µm filter to get rid of any remaining potentially live bacteria. Every vaccine vial was assembled by lyophilizing 1 mL-secretome.

### 2.3. Immunofluorescence

Untreated and Salmonella-infected osteosarcoma primary cells were plated at a concentration of 200,000 cells/mL on polarized microscope slides treated with poly-D-lysine in sterile plates to avoid contamination. After 4 h, samples were washed with PBS and fixed in 4% PFA for 20 min. Cells were rehydrated and blocked with 0.1 M Tris-HCl pH 7.4, 2% FBS, and 0.3% Triton X-100. Staining was performed using mouse anti-connexin43 (1:100, Invitrogen, 138300) and goat anti-lipopolysaccharide (1:100, Antibodies-online, ABIN479062) antibodies overnight. Samples were then washed with 0.1 M Tris-HCl pH 7.4, 0.5% Tween20, and stained with donkey anti-mouse-AF647-conjugate (Thermofisher, Monza, Italy, A21202), donkey anti-goat-AF488-conjugaded antibodies (Thermofisher, A11055), and rabbit anti-alpha smooth muscle Actine-AF555-conjugated antibody (Abcam, Milan, Italy, ABIN479062). Nuclei were counterstained with DAPI (1:45,000, Thermofisher, 1306). Sections were mounted with Vectashield mounting medium and analyzed with a Leica TCS SP8 Laser scanning confocal microscope HC PL APO CS2 40X/1.30 oil immersion objective (Leica). All images were analyzed with Fiji (ImageJ) software version 2.3.0.

### 2.4. ATP Release

Under redox changes and phosphorylation, Cx43 undergoes post-translational modifications, which lead to the opening of hemichannels and the release of intracellular molecules, including ATP, in the surrounding medium [16].

Hence, to assess Cx43 hemichannels opening upon Salmonella infection, we monitored ATP release by measuring ATP through a Cell Titer-Glo Luminescent Cell Viability Assay (Promega, Milan, Italy, G7572). Briefly, supernatants were distributed in 96-well plates and then rapidly mixed with an equivalent volume of MIX-assay solution. Light emission was detected by a luminometer.

### 2.5. Vaccine Administration

One week following the third carboplatin administration, provided that the re-staging did not document distant metastasis, dogs were vaccinated on a monthly basis for a total of 6 dermal injections. Further vaccine doses were allowed on compassionate grounds in the case of metastasis development.

For the first and the second immunizations, the lyophilized vaccine was dissolved in Nobivac Lepto (MSD Animal Health—used as adjuvant), whereas the remaining 4 doses were diluted in sterile water.

Before each vaccination, imiquimod cream (Aldara 5% cream- a TLR7/8 agonist) was rubbed topically on a 3 × 3 cm shaved and clean area as an additional adjuvant. After 15 min, the vaccine was administered intradermally.

### 2.6. Immunomonitoring

In order to monitor the dogs’ immune response, we tested the vaccine-specific T-cell response (from patients-derived peripheral blood mononuclear cells, PBMCs) and the tumor-specific humoral response (from patients-derived serum).

A total of 9 mL of whole blood and 5 mL of serum were collected over 4 sampling timepoints across the 6 vaccinations, namely before the first, second, third, and sixth vaccine administration. The venous blood samples were immediately processed for PBMCs isolation and then stored in a liquid nitrogen tank. Serum was stored at −80 °C.

### 2.7. Vaccine-Specific T-Cell Response

PBMCs collected before and at the last vaccination were tested for their ability to release IFNγ upon stimulation with the vaccine.

Dog PBMCs were thawed, counted, and resuspended in Roswell Park Memorial Institute (RPMI); supplemented with 10% FBS, 2 mM glutamine, and 1x penicillin–streptomycin solution (complete medium), and plated into a 96-well flat bottom plates in the presence of 1 μg/mL of ConA (Sigma, C2010). IL-2 (100 U/mL, Proleukin, Clinigen Healthcare B.V.) was added every other day. After 7–10 days, cells were washed twice and plated in a 96-well plate, 2 × 10^5^ cells/well, with and without the vaccine (the same was used to immunize dogs).

IFN-γ released by the cells was measured after 72 h by ELISA (canine IFN-γ, R&D, CAIF00).

### 2.8. Humoral Response

A primary osteosarcoma cells lysate was generated by freeze and thaw protocol. Lysate derived from 8 × 10^4^ cells was used to coat a well of ELISA plate, overnight at 4 °C. Plates were then left for 2 h at room temperature with 5% BSA solution. Different serum dilutions were incubated on top of the cell lysate for 2 h and the antibody response was measured by adding HRP-conjugated anti-canine IgG antibody (Jackson ImmunoResearch, West Grove, PA, USA).

### 2.9. Control Population

Dogs treated at the authors’ institutions before the vaccine was available and that underwent SOC, consisting of limb amputation and 4–6 cycles of adjuvant carboplatin at the same dosage (i.e., 300 mg/m^2^ IV every 3 weeks), and that were free of pulmonary metastasis at the end of chemotherapy were used as historical controls [17]. Cases were selected starting from 2017 to allow for a period of up to 3 years’ clinical follow-up for any given case.

It was planned to compare treatment outcomes in the current study to the historical group that did not receive immunotherapy.

### 2.10. Antitumor Response Assessment and Follow-Up

Disease status was ascertained by radiologic imaging using standard techniques on a monthly basis for 6 months for vaccinated dogs and every 2 months thereafter, as well as every 2–3 months for dogs receiving SOC until documentation of progressive disease. When clinically indicated, additional diagnostics were performed.

### 2.11. Toxicity

Toxicities related to chemotherapy and vaccination were graded based on the VCOG criteria [18]. All dogs receiving at least one vaccine were evaluable for toxicity.

### 2.12. Sample Size Calculation

In the trial design phase, a sample size calculation was performed to estimate the number of dogs needed to detect a difference in time to metastasis (TTM) of 4 months (SOC: 227 days, SOC + VAX: 347 days) using a two-sided log-rank test at 80% power and at a significance level of 0.05. A sample size of 16 cases and 32 controls was considered adequate. Based upon tolerability and suggested clinical benefits, final accrual ultimately continued to 20 dogs.

### 2.13. Statistical Analysis

Descriptive statistics were used in the analysis of dogs and tumor characteristics. When appropriate, data sets were tested for normality by use of the D’Agostino and Pearson omnibus normality test. Values were expressed as mean ± SD in the case of normal distribution, or as median with a range in the case of non-normal distribution.

Background information recorded for each dog included the following: signalment (i.e., breed, age, sex, weight), tumor anatomic location, type of imaging performed, serum alkaline phosphatase (ALP) activity, blood lymphocyte and monocyte count, tumor histotype, and time between amputation and first day of chemotherapy.

The distribution of demographic features and possible prognostic variables between cases (SOC+VAX group) and controls (SOC group) were assessed with the Mann–Whitney U test or the chi-square test/ Fisher’s exact test for continuous and categorical variables, respectively.

Time to metastasis (TTM) was calculated from the date of amputation to the occurrence of metastatic disease or to the last follow-up visit in which physical examination and thoracic imagine were carried out. Tumor-specific survival (TSS) was calculated from the date of surgery to the date of death for tumor-related causes or to the last visit. If metastasis development or death of tumor-related causes did not occur, dogs were censored for the respective statistical analysis.

Survival plots were generated according to the Kaplan–Meier product-limit method. TTM and TSS were compared between the two groups by means of the log-rank test. One- and two-year survival rates were also calculated for each group.

The influence of other potential prognostic variables on tumor progression and tumor-related death was investigated with univariable and multivariable Cox proportional hazards models. Only covariates with a *p* value < 0.1 at univariable analysis were included in the multivariable (adjusted) regression model.

The following variables were considered: sex, age (7–10 years, other) [19], weight (≥45 kg, other), tumor anatomic location (proximal humerus, other) [19], types of imaging (TBCT, other), serum ALP activity (normal, increased) [19], monocytosis (present, absent) [20], lymphocytosis (present, absent) [21], tumor histotype (chondroblastic, other) [21], and time between amputation and chemotherapy (≤5 days, >5 days) [22].

The correlation between humoral immune response and TSS was also assessed. Patients were given a score (from 0 to 5) based on their antitumor humoral response and a score (from 0 to 5) based on their T-cell response. The two scores were then combined.

Data were analyzed by the use of commercial software programs (SPSS Statistics v. 26, IBM, Somers, NY, and Prism v. 5.0, GraphPad, San Diego, CA); *p* values ≤ 0.05 were considered significant.

Primary objectives sought to assess TTM, TTS, and 1- and 2-year survival rates.

Secondary objectives were to assess feasibility, describe toxicities of the regimen, and determine whether the regimen induced immune responses as measured by T-cell and humoral immune responses.

## 3. Results

### 3.1. Vaccine Preparation and Quality Control

Dogs received either an autologous (derived from their own tumor cells) or heterologous (which was prepared by using osteosarcoma cells of another dog) vaccine.

**Figure 1:**
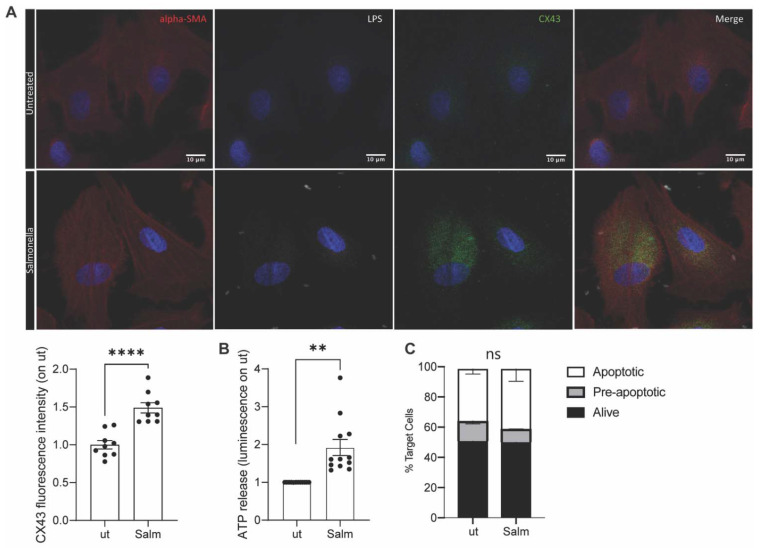
Vaccine quality control. OSA primary tumor cells were infected with Salmonella or left untreated. (**A**). 4 h after infection, cells were fixed for immunofluorescence (IF) analysis. Cell structure was marked with alpha-SMA antibody (red), Salmonella with LPS-specific antibody (white), and hemichannels with Cx43 antibody (green). (**B**). ATP accumulated in cells supernatant was measured after infection. (*n* = 12) (**C**). Frequency of Annexin-PI- (live), Annexin+PI- (early apoptotic), and Annexin+PI+(apoptotic) tumor cells Salmonella-infected (Salm) or untreated (ut) (*n* = 2). Data are represented as mean ± SEM using a scatter dot plot. Statistical analysis was evaluated using two-sided Mann–Whitney test ** *p* < 0.01, **** *p* < 0.0001. scale bar: 10 µm and magnification: 63×.

The upregulation of Cx43 and subsequent opening of the gap junction hemichannels is fundamental to obtain peptide release in the secretome [11]. Thus, we evaluated the upregulation of Cx43 in primary dog osteosarcoma tumor cells upon Salmonella infection. As shown in Figure 1A, Salmonella infection prompted the upregulation and opening of Cx43 hemichannels; indeed, ATP release by tumor cells was observed following the bacterial stimulus (Figure 1B). In agreement with previous studies on human tumor cells, Salmonella treatment did not induce canine tumor cells’ death (Figure 1C), also confirming in the dog system that the vaccine is not a cell lysate.

Every batch of vaccine was qualitatively controlled; if one of the parameters was not met, that particular vaccine batch was excluded.

### 3.2. Patient and Tumor Characteristics

From October 2017 to October 2020, 20 dogs fulfilled the eligibility criteria and were enrolled into the vaccination trial. Eight dogs have been previously described [12]. All dogs underwent limb amputation and received four doses of carboplatin at the dosage of 300 mg/m^2^ IV every three weeks. They were confirmed to be free of pulmonary and abdominal metastatic disease at the third cycle of carboplatin, one week prior to administration of the first vaccine dose. Demographic information and tumor location of these dogs are provided in Supplementary Table S1.

The age, breed, sex, tumor location, histological subtype, lymph node status, type of imaging, serum ALP activity, presence of monocytosis and lymphocytosis, and timing between amputation and initiation of chemotherapy were recorded (Supplementary Table S2).

A control group of 34 dogs treated at the University of Bologna that had undergone amputation and chemotherapy and were confirmed to be free of pulmonary metastatic disease following chemotherapy were retrospectively evaluated (Supplementary Table S1).

There were no significant differences in demographics and prognostic variables between the vaccinated dogs and the control group (Supplementary Table S2).

### 3.3. Chemotherapy

All dogs in either group (vaccinated, SOC+VAX vs. control, SOC) completed the chemotherapeutic protocol. The median time between amputation and start of chemotherapy was 6 days (range, 1–42) for SOC+VAX dogs and 12 days (range, 2–40) for SOC dogs.

All SOC+VAX dogs received a total of 4 carboplatin cycles. With regards to SOC dogs, 19 animals received 4 cycles of carboplatin, 3 dogs received 5 cycles, and 12 dogs received 6 cycles.

Overall, chemotherapy was well tolerated. SOC+VAX dogs experienced 3 episodes of grade 1 bone marrow toxicity, and 1 episode of grade 2 bone marrow toxicity. SOC dogs experienced 3 episodes of grade 1 bone marrow toxicity, and 1 episode of grade 1 gastrointestinal toxicity.

### 3.4. Vaccine Administration

Of the 20 vaccinated dogs, 5 received an autologous vaccine and 15 received a heterologous vaccine.

The median number of vaccines received was 6 (range 3–16). Overall, 10 dogs received all 6 planned vaccinations. Among the remaining 10 dogs, 6 received 5 vaccinations, 3 received 4 vaccinations, and 1 dog received 3 vaccinations. In 7 of them, the reason for not completing the vaccination protocol was disease progression and tumor-related death, whereas 3 dogs died during the protocol for tumor-unrelated causes.

A total of 4 dogs were given prolonged vaccination on compassionate grounds, as there was a possibility that they had had a benefit from the vaccine. They continued intradermal vaccinations on a 2–3 monthly basis and received 10, 11, 13, and 16 vaccinations, respectively.

All dogs were evaluated for toxicity, having received at least one dose of vaccine. In total 131 vaccine doses were administered without any recorded toxicity.

### 3.5. Immunomonitoring

The humoral and T-cell responses were tested for 17 and 14 dogs, respectively. Immunomonitoring was not completed for all dogs, either because some samples could not be taken during the COVID pandemic or because T-cells could not be successfully expanded in vitro.

**Figure 2:**
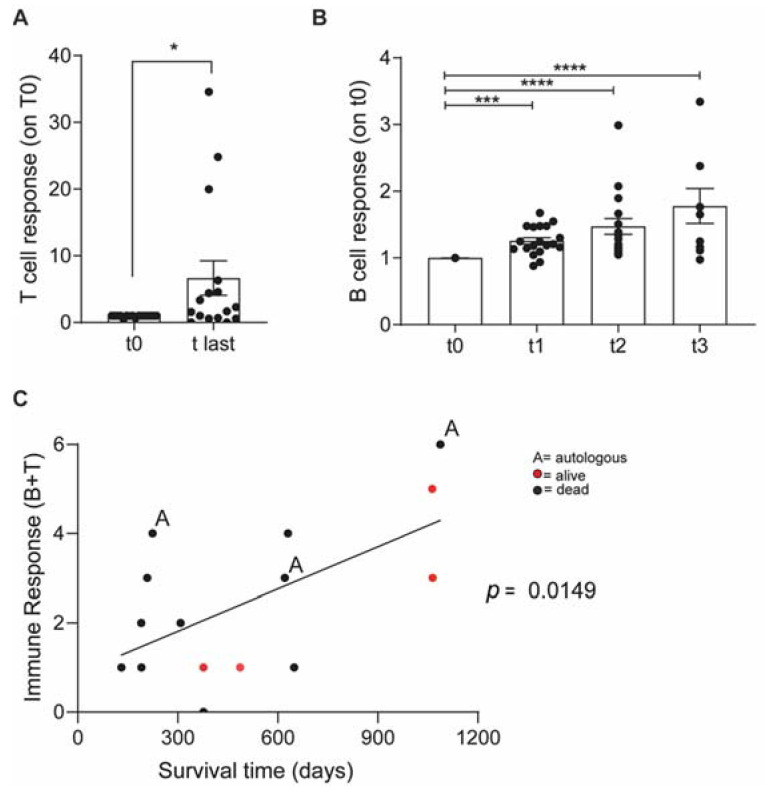
Vaccinated OSA patients developed a long-term vaccine specific antitumor immune response. (**A**)—ELISA quantification of IFNγ released by dog patients PBMCs before vaccination (t0) and at the last vaccination (t-last) upon stimulation with vaccine (Vax). Data are shown as normalized on t0. (**B**)—ELISA quantification of tumor-specific IgG in dog sera. Immunomonitoring experiments were performed once; each condition was tested at least in triplicate. Data are represented as mean ± SEM using a scatter dot plot. Statistical analysis was evaluated using (**A**) two-sided Wilcoxon test. (**B**) One-way ANOVA Kruskal Wallis multiple comparison, * *p* < 0.05, *** *p* < 0.001, **** *p* < 0.0001. (**C**) Correlation Graph: Immune response level and survival.

A significant vaccine-specific immune response was detected (Figure 2A). Vaccinated dogs also developed a tumor-specific immune response and circulating tumor-specific antibodies increased consistently with every vaccination (Figure 2B).

Dogs were then clustered into 6 levels of immune-responsiveness according to the magnitude of both humoral and T-cell responses. The immune response level correlated with TSS (Figure 2C), indicating that successfully boosting the immune system may predict the long-term outcome.

### 3.6. Clinical Outcome

Of the vaccinated dogs, 12 (60%) developed metastatic disease to lungs (*n* = 9), bones (*n* = 2), and lungs, liver and intestine (*n* = 1). In the control group, all (100%) dogs developed pulmonary metastases.

**Figure 3:**
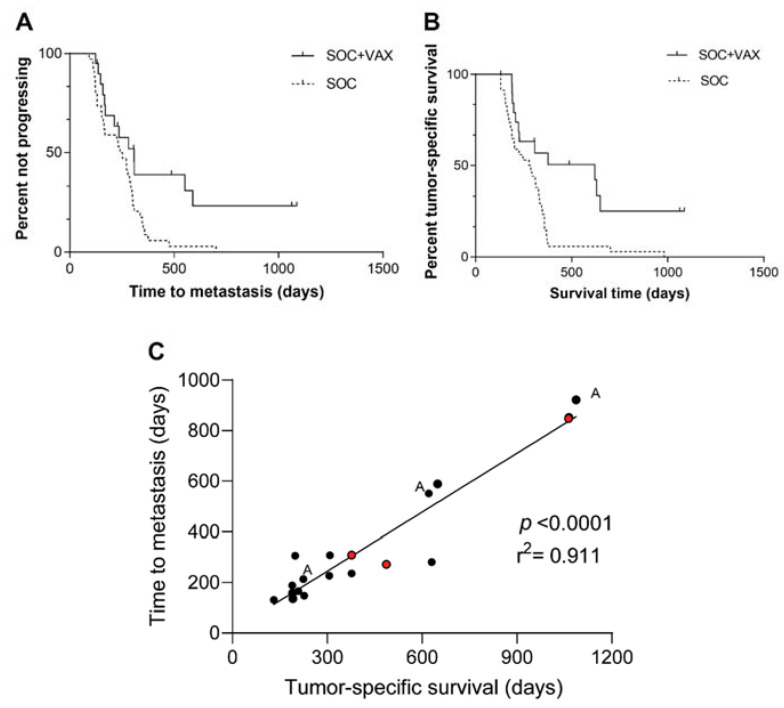
(**A**) Time to metastasis in 54 dogs with non-metastatic appendicular osteosarcoma treated with amputation and chemotherapy, stratified according to whether they received (SOC+VAX, solid line) or did not receive (SOC, dashed line) adjuvant peptide-based vaccination (*p* = 0.010). (**B**) Tumor-specific survival in 54 dogs with non-metastatic appendicular osteosarcoma treated with amputation and chemotherapy, stratified according to whether they received (SOC+VAX, solid line) or did not receive (SOC, dashed line) adjuvant peptide-based vaccination (*p* = 0.002). (**C**) Correlation graph between TTM and TSS.

The median TTM for all 54 dogs was 270 days (95% CI, 221–319). Median TTM was 308 days (95% CI, 217–399) in the SOC+VAX group and 240 days (95% CI, 175–304) in the SOC group (*p* = 0.010; Figure 3A).

At the end of the study, 4 (20%) SOC+VAX dogs were alive after a median follow-up of 775 days (range, 487–1064) and 16 had died. Cause of death was attributable to osteosarcoma metastasis in 12 dogs (75%) and to unrelated causes in the remaining 4 (25%) dogs. Necropsy ruled out osteosarcoma in these 4 dogs; cause of death was attributable to intestinal volvulus, gastric dilation/volvulus, severe coxofemoral arthrosis limiting quality of life, and protein-losing enteropathy. Overall survival rates at 1 and 2 years were 53% and 20%, respectively.

All SOC dogs had died due to osteosarcoma metastasis. One- and 2-year survival rates were 15% and 3%, respectively.

The median TSS of all 54 dogs was 308 days (95% CI, 232–384). Median TSS was 621 days (95% CI, 98–1144) in the vaccine group and 278 days (95% CI, 181–375) in the control group. This difference was significant (*p* = 0.002; Figure 3B).

The median time between the diagnosis of metastasis and death was 53 days (range, 1–349) for SOC+VAX dogs and 19 days (range, 1–507) for SOC dogs (*p* = 0.040).

Finally, TTM significantly correlated with TSS (*p* < 0.0001; Figure 3C).

Results from univariable and multivariable analysis are presented in Table 1 and Table 2. On univariable analysis, variables associated with both an increased risk of tumor progression and tumor-related death were increased ALP activity and lack of vaccine administration. On multivariable analysis, both variables retained prognostic significance for TSS, whereas only lack of vaccine administration was significantly associated with an increased risk of tumor metastases.

## 4. Discussions

Dogs with osteosarcoma have poor prognosis. Despite research, no substantial improvement has been achieved in the therapy of these patients in the last decades, and long-term survival is still <25% [1,2].

Here, we report the results of a target animal effectiveness study that combines SOC with a peptide-based anticancer vaccine in dogs with non-metastatic appendicular osteosarcoma. The concept that osteosarcoma is a “hot” tumor has only recently become clear. Hot tumors are characterized by infiltration of immune cells and have a proinflammatory milieu, thus, being more responsive to therapeutic vaccination [23]. Several clinical trials have shown encouraging clinical and immunologic response using immunotherapy in dogs with osteosarcoma [11,24,25], thus, paving the path for further investigations [26], moreover, in view of the fact that SOC has not appreciably changed in several decades.

We have previously reported that infection of tumor cells with Salmonella induces the opening of Cx43-gap junctions and the transfer of antigenic peptides between adjacent cells [27,28]. Further study has shown that the same stimulus triggered by Salmonella can induce the extracellular release of proteasome-generated peptides by unopposed Cx43 hemichannels via the exacerbation of ER stress and the unfolded protein response pathway [12]. We showed that ERstrePs released by tumor cells can induce an antitumor response when used as a vaccine formulation in several animal models [12]. Here, we translated these preclinical findings in a target animal effectiveness trial in dogs with spontaneously occurring osteosarcoma. We documented that our vaccination strategy was able to increase TTM, thereby prolonging survival while maintaining a safe profile. Additionally, vaccinated dogs developed a long-term specific antitumor response, as documented by the immunomonitoring of these patients over time.

Numerous works have shown that therapeutic cancer vaccines stimulate the patient’s adaptive immune system against specific tumor antigens to regain control over tumor growth and eradicate minimal residual disease, including micrometastases [29]. The basic principles that are necessary for successful therapeutic vaccination include (1) low disease burden; (2) limited immunosuppression, as obtained with certain chemotherapeutic drugs; (3) delivery of large amounts of high-quality antigens to dendritic cells; (4) optimal dendritic cell activation; (5) induction of strong CD4+ T helper cell and cytotoxic T-lymphocyte responses; and (6) long-lasting immunity [30,31,32,33].

In the current study, these principles were matched, as documented by the dogs’ clinical outcome and immune response.

To reduce tumor burden, dogs were first amputated and subsequently treated with carboplatin. Contrary to common belief, some chemotherapy drugs, including platinum compounds, are able to promote immunity by making tumor cells more immunogenic and sensitive to lysis [34,35]. There is evidence that the efficacy of some conventional chemotherapeutics, including platinum compounds, involves not only their cytotoxic effect, but also relies on modulation of the immune system by reducing the numbers of immunosuppressive myeloid cells [36]. Additionally, tumor cell death triggers a clinically significant immunogenic response [37,38,39]. All types of cancer vaccines stand to benefit from chemotherapy combinations [40]; in this trial, vaccination was started during chemotherapy, yet it did not prevent the mounting of a significant immune response.

To date, most cancer vaccines have targeted tumor-associated antigens, which are self-proteins that are abnormally expressed by cancer cells, leading to immune tolerance and eventually to treatment failure.

Another strategy relies on the use of neoantigens, which can elicit a strong antitumor response. However, identification and use of the latter cannot be applied on a large scale as they require a personalized approach.

Our peptide-based vaccine is composed by ERstrePs, which are shared non-mutated tumor antigens expressed only by ER-stressed tumor cells but not by healthy (non-ER stressed) cells, and that can boost a strong antitumor response [12]. Since peptidomic analysis was not performed, the presence of neoantigens in the vaccine formulation cannot be excluded.

To obtain ERstrePs, tumor cells were infected with Salmonella, and the secretome was then used as a vaccine. During the initial phase of the trial, some dogs received the autologous vaccine, but over time the choice was made to vaccinate the other patients with a heterologous vaccine, the rational of which relies in the observation that ERstrePs are shared, and therefore, they are potentially applicable to all dogs with no need for an autologous vaccination strategy, eventually saving costs and time [12].

Contrary to autologous vaccines, the use of heterologous vaccines has numerous advantages. First, surgical specimens may not always be available to produce the vaccine or cells may not be expanded in vitro. Second, the preparation of an autologous vaccine is time-consuming and expensive. Third, an off-the-shelf vaccine can be administered immediately to all dogs in need, thus, reducing the time to treatment. Due to the above, once feasibility, safety, and efficacy of the heterologous approach were confirmed [12], this became our first option to guarantee a rapid availability of the vaccine for all dogs.

The first and second vaccine doses were dissolved in Nobivac Lepto, which was used as an adjuvant to induce a CD4+ immune response. Dogs are routinely vaccinated against Leptospira; thus, we primed the immune response against the known Leptospira antigens to enhance the anticancer immune response [41,42]. Imiquimod, a Toll-like receptor 7/8 agonist, was rubbed topically before each vaccination to enhance the immune response, as described in human immunotherapy trials [43].

Overall, vaccination was well tolerated, with no side effects reported even in the four dogs that were given compassionate permission to continue being immunized for more than a one year period.

To verify the capability of the vaccine to elicit an antitumor immune response, dogs underwent immunomonitoring at baseline (pre-vaccination) and at three timepoints during vaccination, which showed the induction of strong humoral and cellular immune responses. This finding mirrors the clinical data and shows that the stronger the elicited immune response, the longer the survival. The therapeutic effect of the vaccine was evaluated by comparing vaccinated dogs with a historical and well-balanced population of dogs with the same disease. Vaccinated dogs had a significantly longer survival time compared with dogs undergoing SOC (621 vs. 278 days, respectively), with a 1- and 2-year survival rate of 53% and 20%, respectively, compared with 15% and 3%. Vaccinated dogs also experienced a significantly longer TTM compared with unvaccinated animals (308 vs. 240 days, respectively). The outcome in terms of TTM and TSS of SOC-treated unvaccinated animals is similar to the one reported elsewhere, which ranged from 123–257 days and 207–321 days, respectively [21,40,44,45,46,47,48,49]. Although the 1-year survival rate of unvaccinated dogs is similar to that of 16–20% previously reported [2,46], it appears disappointingly low compared with other studies. There are possible explanations for the poor outcome documented in our control population. First, none of the dogs included in the control group underwent rescue treatment once metastatic disease was detected. With the exception of three dogs, euthanasia was carried out within 3 months from the diagnosis of pulmonary metastasis. It may be possible that this accounted for the poor outcome in this particular control group. Indeed, in some of the previous papers, or at least in those in which it is declared, dogs developing metastatic disease were subjected to rescue treatments, possibly impacting survival time. Second, in our control group, one fourth of dogs had a humeral osteosarcoma, which may have impacted prognosis.

It is expected that immune memory, eliciting a long-lasting immunity against tumor recurrence, will be generated in response to immunotherapies. In our trial, 12 vaccinated dogs died during the study because of metastatic disease. Notably, a long interval was documented between the clinical onset of distant metastasis and cancer-related euthanasia, which was significantly different from dogs undergoing SOC. Although it may be possible that owners enrolling their animals into clinical trials are more motivated to keep them alive despite metastatic disease, all vaccinated dogs in this series maintained good quality of life and did not undergo any rescue treatment, thereby suggesting that metastases may grow more slowly in vaccinated dogs than in dogs undergoing SOC, presumably due to the establishment of a long-lasting immunological memory.

There are some limitations in this study.

To shorten the study, all entering dogs with newly diagnosed osteosarcoma could be assigned to the immunotherapeutic trial, and a historical population of dogs undergoing SOC at one of the authors’ institutions was used as control. Vaccinated and unvaccinated dogs were well balanced concerning any known or putative prognostic factor, thereby ensuring comparability of the treatment arms.

However, it must be acknowledged that restaging was not standardized for unvaccinated dogs, however, this did not impact our outcome data. On the contrary, vaccinated dogs were monitored more frequently than dogs receiving SOC, therefore, metastatic disease would have been recognized earlier, thereby shortening TTM. Finally, dogs were included in the control group if free from pulmonary metastases at the end of chemotherapy cycles, whereas vaccinated dogs were included upon absence of metastatic disease at the third carboplatin administration, thereby potentially selecting a control population of dogs with a better outcome/response to chemotherapy. Overall, in all cases, if there was a difference, outcome data would not be biased in favor of vaccinated dogs.

In the current trial, neoplastic cells were derived from canine donors, either in the autologous or in the heterologous setting. We are now exploring the possibility of using a mixture of cells, originating from different donors and different tissues (primary and metastatic), aimed at broadening the target spectrum for activated CD8+ T-cells.

We initially planned six total vaccinations for each dog; however, four dogs received prolonged vaccination on compassionate grounds, with no reported toxicity. If and how frequently a dog should be boosted remains to be defined. It would be interesting to dissect out the contribution of humoral or cellular immune responses in the observed clinical benefit, as both were initiated in response to vaccination.

## 5. Conclusions

To conclude, osteosarcoma is a fatal disease both in humans and dogs, and new strategies beside surgery and chemotherapy are warranted to improve long-term outcome. Harnessing the immune system is an attractive way to potentially increase survival, as immunotherapy has been substantiated by studies on osteosarcoma immunogenicity. By using a bacterial-based strategy for vaccine development, we report the induction of an efficacious immune response, ultimately translating into improved clinical outcome. The results of this clinical trial provide promising potential for future management in both humans and dogs.

## Figures and Tables

**Table 1:** Univariable Cox regression analysis of variables potentially associated with increased risk of tumor metastasis and tumor-specific survival in 54 dogs with non-metastatic appendicular osteosarcoma treated with amputation and chemotherapy.

Variable	Time to Metastasis	*p*	Tumor-Specific Survival	*p*
Age <7 or >10 years	0.82 (0.46–1.46)	0.503	0.90 (0.50–1.62)	0.733
Giant breed (≥45 kg)	0.66 (0.26–1.67)	0.382	0.55 (0.20–1.53)	0.253
Male sex	1.28 (0.72–2.28)	0.408	1.47 (0.82–2.64)	0.197
Tumor located in proximal humerus	1.13 (0.59–2.15)	0.712	1.39 (0.73–2.66)	0.322
Increased serum ALP activity	2.54 (1.02–6.32)	0.045 *	2.85 (1.12–7.24)	0.027 *
Chondroblastic histotype	0.41 (0.10–1.69)	0.216	0.39 (0.09–1.60)	0.188
>5 days between amputation and chemotherapy	1.47 (0.80–2.70)	0.216	1.72 (0.92–3.21)	0.087
Lack of vaccine administration	2.33 (1.20–4.50)	0.012 *	2.80 (1.42–5.54)	0.003 *

* significant.

**Table 2:** Multivariable Cox regression analysis of variables potentially associated with increased risk of tumor metastasis and tumor-specific survival in 54 dogs with non-metastatic appendicular osteosarcoma treated with amputation and chemotherapy.

Variable	Time to Metastasis	*p*	Tumor Specific Survival	*p*
Increased serum ALP activity	2.30 (0.92–5.72)	0.073	2.67 (1.05–6.76)	0.039 *
Lack of vaccine administration	2.25 (1.16–4.37)	0.017 *	2.75 (1.39–5.44)	0.004 *

* significant.

## Data Availability

The data presented in this study are available on request from the corresponding author. The data are not publicly available due to privacy restrictions.

## Supplementary Materials

The following supporting information can be downloaded at: https://www.mdpi.com/article/10.3390/cancers14051347/s1, Table S1: Demographic information and tumor location in 54 dogs with non-metastatic appendicular osteosarcoma treated with amputation and chemotherapy, stratified according to whether they received (SOC+VAX) or did not receive (SOC) adjuvant peptide-based vaccination; Table S2: Distribution of variables potentially associated with prognosis in 54 dogs with non-metastatic appendicular osteosarcoma treated with amputation and chemotherapy, stratified according to whether they received (SOC+VAX) or did not receive (SOC) adjuvant peptide-based vaccination.
