# Supplementary material for: A Target Animal Effectiveness Study on Adjuvant Peptide-Based Vaccination in Dogs with Non-Metastatic Appendicular Osteosarcoma Undergoing Amputation and Chemotherapy
Source: Cancers (Basel). 2022 Mar 6;14(5):1347. doi: 10.3390/cancers14051347 (PMC8909565; doi:10.3390/cancers14051347)
Supplement: Supplementary file 1 [file cancers-14-01347-s001.zip › cancers-1605943-supplementary.pdf]

Supplementary Materials: A Target Animal Effectiveness Study on Adjuvant Peptide-Based Vaccination in Dogs with Non-Metastatic Appendicular Osteosarcoma Undergoing Amputation and Chemotherapy

Laura Marconato, Alessia Melacarne, Marina Aralla, Silvia Sabattini, Luca Tiraboschi, Valentina Ferrari, Offer Zeira, Andrea Balboni, Eugenio Faroni, Dina Guerra, Luciano Pisoni, Erica Ghezzi, Letizia Pettinari and Maria Rescigno

Table S1. Demographic information and tumor location in 54 dogs with non-metastatic appendicular osteosarcoma treated with amputation and chemotherapy, stratified according to whether they received (SOC+VAX) or did not receive (SOC) adjuvant peptide-based vaccination.

	**SOC+VAX (n = 20)**	**SOC (n = 34)**
Median age (range)	8 (3-12) years	7 (1-13) years
Purebred dogs	16	22
Most represented breeds	Boxer (n = 3); Rottweiler (n = 3); Leonberger (n =2)	Rottweiler (n = 6); German shepherd (n = 4); Labrador retriever (n = 2)
Median weight (range)	35 (14-72) kg	32 (11-63) kg
Sex	8 males; 12 females	18 males; 16 females
Tumor location	scapula (n = 1); proxymal humerus (n = 5); distal radius (n = 4); distal ulna (n = 2); distal femur (n = 3); proximal tibia (n = 4); distal tibia (n = 1)	scapula (n = 2); proxymal humerus (n = 9); distal humerus (n = 1); distal radius (n = 8); proxymal femur (n = 2); distal femur (n = 7); proximal tibia (n = 5)

Table S2. Distribution of variables potentially associated with prognosis in 54 dogs with non-metastatic appendicular osteosarcoma treated with amputation and chemotherapy, stratified according to whether they received (SOC+VAX) or did not receive (SOC) adjuvant peptide-based vaccination.

**Variable**	**SOC+VAX (n = 20)**	**SOC (n = 34)**	**P**
Age <7 or >10 years			
yes	9	18	0.573
no	11	16	
Giant breed (≥45 kg)			
yes	4	3	0.238
no	16	31	
Sex			
male	8	18	0.358
female	12	16	
Tumor location			0.905
proximal humerus	5	9	

other	15	25	
Type of imaging			
TBCT	14	20	0.411
other	6	14	
Serum ALP activity			
normal	2	5	0.619
increased	18	29	
Monocytosis			
present	0	0	NA
absent	20	34	
Lymphocytosis			
present	0	0	NA
absent	20	34	
Histotype			
chondroblastic	3	1	0.102
other	17	33	
Time between amputation and chemotherapy			0.130
≤5 days	10	24	
>5 days	10	10	
